# Identification of cancer-specific motifs in mimotope profiles of serum antibody repertoire

**DOI:** 10.1186/s12859-017-1661-5

**Published:** 2017-06-07

**Authors:** Ekaterina Gerasimov, Alex Zelikovsky, Ion Măndoiu, Yurij Ionov

**Affiliations:** 1 Department of Computer Science, Georgia State University, 25 Park Place, Atlanta, 30303 GA USA; 2 Department of Computer Science and Engineering, University of Connecticut, Storrs, 06269 CT USA; 3 Department of Cancer Genetics, Roswell Park Cancer Institute, Buffalo, 14263 NY USA

**Keywords:** Random peptide phage display library, Early cancer detection, Immune response, Peptide motifs, Mimotope profile

## Abstract

**Background:**

For fighting cancer, earlier detection is crucial. Circulating auto-antibodies produced by the patient’s own immune system after exposure to cancer proteins are promising bio-markers for the early detection of cancer. Since an antibody recognizes not the whole antigen but 4–7 critical amino acids within the antigenic determinant (epitope), the whole proteome can be represented by a random peptide phage display library. This opens the possibility to develop an early cancer detection test based on a set of peptide sequences identified by comparing cancer patients’ and healthy donors’ global peptide profiles of antibody specificities.

**Results:**

Due to the enormously large number of peptide sequences contained in global peptide profiles generated by next generation sequencing, the large number of cancer and control sera is required to identify cancer-specific peptides with high degree of statistical significance. To decrease the number of peptides in profiles generated by nextgen sequencing without losing cancer-specific sequences we used for generation of profiles the phage library enriched by panning on the pool of cancer sera. To further decrease the complexity of profiles we used computational methods for transforming a list of peptides constituting the mimotope profiles to the list motifs formed by similar peptide sequences.

**Conclusion:**

We have shown that the amino-acid order is meaningful in mimotope motifs since they contain significantly more peptides than motifs among peptides where amino-acids are randomly permuted. Also the single sample motifs significantly differ from motifs in peptides drawn from multiple samples. Finally, multiple cancer-specific motifs have been identified.

## Background

Circulating autoantibodies produced by the patient’s own immune system after exposure to cancer proteins are promising biomarkers for the early detection of cancer. It has been demonstrated, that panels of antibody reactivities can be used for detecting cancer with high sensitivity and specificity [1].

The whole proteome can be represented by random peptide phage display libraries (RPPDL). For any antibody the peptide motif representing the best binder can be selected from the RPPDL. The next generation (next-gen) sequencing technology makes possible to identify all the epitopes recognized by all antibodies contained in the human serum using one run of the sequencing machine.

Recent studies tested whether immunosignatures correspond to clinical classifications of disease using samples from people with brain tumors [2]. The immunosignaturing platform distinguished not only brain cancer from controls, but also pathologically important features about the tumor including type and grade. These results clearly demonstrate that random peptide arrays can be applied to profiling serum antibody repertoires for detection of cancer.

In [3] the authors studied serum samples from patients with severe peanut allergy using phage display. The phages were selected based on their interaction with patient serum and characterised by highthroughput sequencing. The epitopes of a prominent peanut allergen, Ara h 1, in sera from patients could be identified.

The profiles generated by next-gen sequencing following several iterative round of affinity selection and amplification in bacteria can consist of millions of peptide sequences. A significant fraction of these sequences is not related to the repertoires of antibody specificities, but produced by nonspecific binding and preferential amplification in bacteria. The presence of high amounts of these unspecific, quickly growing "parasitic" sequences can complicate the analysis of serum antibody specificities. Considering that the affinity selected sequences can be clustered into the groups of similar sequences with shared consensus motifs, while the parasitic sequences are usually represented by single copies, we propose a novel motif identification method (CMIM) based on CAST clustering [4].

We have shown that the amino-acid order is meaningful in mimotope motifs found by CMIM – the CMIM motifs identified in observed samples contain significantly more peptides then motifs among the same peptides but with amino-acids randomly permuted. Also the single sample motifs are shown to be significantly different from motifs in peptides drawn from multiple samples.

CMIM was applied to case-control data and identified numerous cancer-specific motifs. Although no motif is statistically significant after adjusting to multiple testing, we have shown that the number of found motifs is much larger than expected and may therefore contain useful cancer markers.

## Methods

### Generating mimotope profiles of serum antibody repertoire

**Fig. 1:**
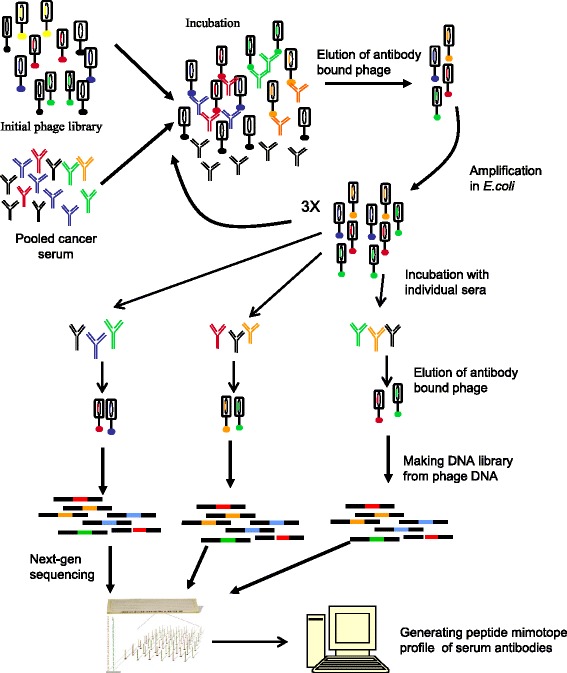
A scheme for generating mimotope profiles of serum antibody repertoire. The first step of the experiment is library enrichment, the second step is directly generating of mimotope profiles and next-gen sequencing

The experiment for generating mimotope profiles of serum antibody repertoire is outlined in the flowchart in Fig. 1. The first step of the experiment was library enrichment, the second step was directly generating of mimotope profiles and next-gen sequencing.

#### Library enrichment

Pooled serum from eight stage 0 breast cancer patients were used for enrichment of the library. The enrichment was performed as follows. Twenty *μ* *l* of pooled serum and 10 *μ* *l* of the Ph.D.7 random peptide library (NEB) were diluted in 200 *μ* *l* of the Tris Buffered Saline (TBST) buffer containing 0.1% Tween 20 and 1% BSA and incubated overnight at room temperature. The phages bound to antibodies were isolated by adding 20 *μ* *l* of protein G agarose beads (Santa Cruz) to the phage –antibody mixture and incubating for 1 hour. To eliminate the unbound phage the mixture with beads was transferred to the well of 96-well MultiScreen-Mesh Filter plate (Millipore) containing 20 *μ* *m* pore size nylon mesh at the bottom. The unbound phage was removed by applying vacuum to the outside of the nylon mesh using micropipette tip. The beads were washed 4 times by adding to the well 100 *μ* *l* of TBST buffer and removing the liquid by applying vacuum to the outside of the nylon mesh using micropipette tip. The phage bound to the antibodies was eluted by adding to the beads of 100 *μ* *l* of 100 *mM* Tris-glycine buffer pH 2.2 followed by neutralization using 20 *μ* *l* 1 M Tris buffer pH 9.1. The eluted phages were amplified in bacteria by infecting 3 *ml* of an early log-phase culture. The amplified phages were isolated by precipitating phage with ^1^/_6_ volume of 20% PEG, 05.M NaCl precipitation buffer. The cycle of incubation-bound phage isolation-amplification was repeated two more times and the isolated after the 3rd amplification library was used for analyzing antibody repertoires.

#### Generating peptide profiles

Twenty *μ* *l* of serum and 10 *μ* *l* of the enriched library were diluted in 200 *μ* *l* of the Tris Buffered Saline (TBST) buffer containing 0.1% Tween 20 and 1% BSA and incubated overnight at room temperature. The phages bound to antibodies were isolated using low pH buffer as described above for the enrichment of the library and the phage DNA was isolated using phenol-chloroform extraction and ethanol precipitation. The 21 *nt* long DNA fragments coding for random peptides were PCR-amplified using primers containing a sequence for annealing to the Illumina flow cell, the sequence complementary to the Illumina sequencing primer and the 4 *nt* barcode sequence for multiplexing. The PCR-amplified DNA library was purified on agarose gemultiplexed and sequenced by 50 cycle HiSeq 2500 platform.

The sequences were de-multiplexed to determine its source sample. The 21- base nucleotides were extracted between base position 29 and 49 and translated to 7-amino-acid peptide using the first frame. Any peptide containing stop codon was discarded.

### CAST-based motif identification method

A motif was defined as a group of peptides having common sequence pattern. If we consider a motif as a cluster formed by peptides with the center represented by a consensus sequence then construction of a motif corresponds to a difficult clustering problem with many closely located centers. The radius of a cluster may exceed the distance from one cluster to another one. To solve the problem we modified CAST clustering algorithm (Clustering Affinity Search Technique) [4]. We did not know in advance how many motifs should be found in each sample. Other words, we did not know the number of clusters. For this reason we used CAST. It does not assume a given number of clusters and an initial spatial structure of them, but determines cluster number and structure based on the data.

The input of CAST consists of a similarity matrix to store the distances of all of the peptides and an similarity threshold. We defined the *similarity* of two sequences of equal length as the number of positions where the corresponding symbols are equal. We also consider the shifts of sequences relative to each other where it is necessary. For example, if we have two peptide sequences MLPHWAS and LPHWASK we need to shift them on one position relative to each other to get common overlap LPHWAS. In this example the similarity will be equal 6. Since the minimal length of a peptide sequence that can mimic the epitope recognized by antibody is usually in the range from 4 to 7 amino acids, we assigned similarity threshold equal 4. So any two peptides in a motif should have approximately 4 common amino acids (diameter of a motif). As well as no more than three shifts between peptides to the right or left sides were allowed.



The Algorithm 1 describes the CAST-based motif identification method (CMIM).

On every iteration of the algorithm two peptides with the highest similarity were chosen as the initial center of a cluster. Next the process of adding and removing of peptides from the cluster was performed while the similarity between every pair of petides in a final set were not less than the threshold. During that step initially assigned central peptides could be removed. A measure of similarity between a peptide and all other peptides in a cluster was called *affinity*. Obtained cluster was saved removing its peptides from further consideration as initial centers. Then the procedure was repeated to find remaining motifs. Unlike CAST our algorithm allows intersection between clusters. As result some consensus sequences of motifs could be too close to each other. So the obtained clusters were collapsed if they had more than 50% common peptides. The last step was to align all peptides in the cluster and compute entropy in every position. Seven positions with the smallest cumulative entropy (the most conserved part) were chosen, and the consensus amino acid sequence was found. The output of the algorithm was a set of finding motifs in a serum sample, each represented by a cluster and its consensus 7-mer sequence. To compute consensus sequence for a motif we aligned peptide sequences in its cluster and calculated entropy in every position of the cluster. Then we chose seven positions window with the minimum total entropy and identified consensus as the order of the most frequent amino acids found at each chosen position.

## Results and discussion

### Data set

We analyzed the profiles generated for the 15 serum samples of the stage 0 and 1 breast cancer patients and for the 15 serum samples of the healthy donors. For each serum sample the experiment was performed separately using the same enriched library on all samples. In average, for the experimental condition selected, the total number of distinct peptide sequences generated in one sample was 18450, and standard deviation *σ* was 6205. The average count value (expression) of a sample was 407335(*σ* = 252393).

After applying the motifs search separately to every sample, we obtained in average 3000(1073) motifs per a control sample and 3490(1315) motifs per a case sample. The average size of a motif in a case was 7.1(1.8) peptides, in a control it was 6.8(1.3) peptides. Every sample contained significant amount of large motifs. Thus, the average number of motifs consisting of 20 and more peptides was 154(71) and 131(53) for cases and controls respectively.

### Motif validation

To validate found motifs we generated pseudo mimotope profiles using two strategies. The first strategy was random permutation of amino acids in a sample peptides. As result, we received 30 samples consisting of random 7-mer peptides. We ran our motif search method on the samples and obtained about 6639(1967) motifs with the average size 4.2(0.7). Although, the largest motif among all samples contained only 17 peptides. More than 95% of motifs in all samples had size no more than 4 peptides.The obtained motifs were significantly different from those found in real serum samples. This result proves the amino-acid order is meaningful in mimotope motifs found by CMIM.

The second strategy was random selection of peptides from existing samples and generating random samples. We collapse all original serum samples together assigning count value to each peptide. The more abundant and popular a peptide was among samples the more probable it would be selected to a new random sample. We generated 30 samples with 20k peptides each. We also applied motif search method to the random samples. In average we obtained 3890(34) motifs with the size of 5.71(0.04) peptides. To compare the group of random samples with the group of real serum samples we applied Kruskal–Wallis test [5]. This non-parametric method determines whether samples originate from the same distribution. The result *p-value* was 7.5∗10^−5^ rejecting the null hypothesis that the population medians of both groups were equal. Thus, the single sample motifs are significantly different from motifs in peptides drawn from multiple samples.

### Cancer-specific motifs

The cancer-specific motifs were defined as motifs significantly prevalent in cases. We compared motifs based on their consensus 7-mers. If two samples shared any consensus sequence, we considered they shared the corresponding motif. A motif was associated with cancer if probability of its appearance in cases against controls by chance was less than 0.05. We calculated the probability of all possible combinations of 15 cases and 15 controls and chose the most discriminating. As result, we received the following case-control significant combinations with probability less 0.05: 4-0 (a motif should appeared in 4 cases and 0 controls), 5-0, 6-0,...,15-0,6-1,...,15-1,8-2,...15-2,9-3,...15-3,10-4,...,15-4,11-5,...15-5,12-6,...,15-6,13-7,...,15-7,14-8,...,15-8,...,15-11. We also found the combinations with probability less than 0.04, 0.03, 0.02 and 0.01. There were 67 cancer specific motifs with probability of case-control appearance less than 0.05, 27 motifs with probability less than 0.04, 24 motifs with probability less than 0.03, 10 and 4 motifs with probability less than 0.02 and 0.01 respectively.

To validate obtained motifs we applied permutation test. We tested, at 5% significance level, whether the number of observed motifs can be obtained by chance. The test proceeded as follows. Cases and controls were randomly swapped, so some cases were considered as controls while controls were considered as cases. Totally 10K random permutations were performed. For every permutation the number of motifs with significant case-control appearance was count. The one-sided *p-value* of the test was calculated as the proportion of permutations where the number of significant motifs was greater or equal to observed number (see Table 1). As far as all p-values were greater than 0.05 we can not reject the hypothesis that the number of observed motifs could be obtained by chance. The number of expected and observed motifs as well as False Discovery Rate (FDR) [6] adjustment are also shown in Table 1. Notice that the number of observed motifs with probability of case-control appearance less than 0.01 equals to 4 which is less than expected number 4.2. That gives FDR greater than 1. Despite the fact that no motif is statistically significant, we can see that their number is still larger than expected.

**Table 1:** Statistics for case-specific motifs

Probability	Observed	Expected	FDR	*p-value* of the permutation test
<0.05	67	51.9	0.77	0.15
<0.04	27	20.5	0.76	0.21
<0.03	24	16.6	0.69	0.15
<0.02	10	8.1	0.81	0.32
<0.01	4	4.2	1.06	0.52

The number of observed motifs with expected number, FDR and *p-value* of the permutation test

## Conclusions

In current work we identified cancer-specific motifs by analyzing peptide profiles of serum samples from cancer patients and from healthy donors. These profiles were generated using a phage DNA sequencing following single selection without amplification on the serum samples with the library enriched by the cycles of affinity selection-amplification using a pool of serum samples from additional cancer patients.

A novel motif identification method based on CAST clustering (CMIM) was proposed. We found that for any real serum sample the number of peptides per a motif is significantly greater comparing with pseudo epitope repertoire consisting of a randomly permuted peptides. Also the single sample motifs are shown to be significantly different from motifs in peptides drawn from multiple samples.

Running on case-control data CMIM identified cancer-specific motifs. Although no motif is statistically significant after permutation test, the number of found motifs is larger than expected and may therefore contain useful cancer markers.
